# The impact of HIV-associated lipodystrophy on healthcare utilization and costs

**DOI:** 10.1186/1742-6405-5-14

**Published:** 2008-07-01

**Authors:** Jeannie S Huang, Karen Becerra, Susan Fernandez, Daniel Lee, WC Mathews

**Affiliations:** 1 Department of Pediatrics, University of California, San Diego, La Jolla, California, USA; 2 Department of Medicine, University of California, San Diego, La Jolla, California, USA

## Abstract

**Background:**

HIV disease itself is associated with increased healthcare utilization and healthcare expenditures. HIV-infected persons with lipodystrophy have been shown to have poor self-perceptions of health. We evaluated whether lipodystrophy in the HIV-infected population was associated with increased utilization of healthcare services and increased healthcare costs.

**Objective:**

To examine utilization of healthcare services and associated costs with respect to presence of lipodystrophy among HIV-infected patients.

**Methods:**

Healthcare utilization and cost of healthcare services were collected from computerized accounting records for participants in a body image study among HIV-infected patients treated at a tertiary care medical center. Lipodystrophy was assessed by physical examination, and effects of lipodystrophy were assessed via body image surveys. Demographic and clinical characteristics were also ascertained. Analysis of healthcare utilization and cost outcomes was performed via between-group analyses. Multivariate modeling was used to determine predictors of healthcare utilization and associated costs.

**Results:**

Of the 181 HIV-infected participants evaluated in the study, 92 (51%) had clinical evidence of HIV-associated lipodystrophy according to physician examination. Total healthcare utilization, as measured by the number of medical center visits over the study period, was notably increased among HIV-infected subjects with lipodystrophy as compared to HIV-infected subjects without lipodystrophy. Similarly, total healthcare expenditures over the study period were $1,718 more for HIV-infected subjects with lipodystrophy than for HIV-infected subjects without lipodystrophy. Multivariate modeling demonstrated strong associations between healthcare utilization and associated costs, and lipodystrophy score as assessed by a clinician. Healthcare utilization and associated costs were not related to body image survey scores among HIV-infected patients with lipodystrophy.

**Conclusion:**

Patients with HIV-associated lipodystrophy demonstrate an increased utilization of healthcare services with associated increased healthcare costs as compared to HIV-infected patients without lipodystrophy. The economic and healthcare service burdens of HIV-associated lipodystrophy are significant and yet remain inadequately addressed by the medical community.

## Background

The HIV-associated lipodystrophy syndrome is characterized by alterations in body appearance related to changes in body fat stores and has been described in up to 80% of persons who have been exposed to antiretroviral therapies [1-4]. These changes in body appearance have been shown to result in body image dysphoria and reduced body image-related quality of life among affected persons [5,6]. In addition, HIV-infected persons with lipodystrophy have reported poorer physical health [7]. Among other patient populations, poorer health perceptions [8] and quality of life [9] have both been associated with increased healthcare costs and utilization. However, little is known about health services use among HIV-infected patients with lipodystrophy.

Health services use is an important indicator of clinical significance because it indicates patient suffering and denotes social and economic burdens due to the explicit and hidden (e.g. time lost from work) costs associated with health services use. Health services use has been evaluated in the HIV-infected population, and HIV disease is associated with elevated health services use [10]. However, the effect of lipodystrophy on health services use and associated healthcare expenditures in this population has yet to be explored. We therefore sought to determine whether patients affected by HIV lipodystrophy exhibited changes in their utilization of healthcare resources as compared to HIV-infected patients without lipodystrophy. Our a priori hypothesis was that HIV-infected patients with lipodystrophy would demonstrate increased utilization of healthcare services with an associated increase in healthcare expenditures as compared to HIV-infected patients without lipodystrophy.

## Results

### Demographics

The demographic data of the one hundred and eighty-one HIV-infected study participants are displayed in [see Additional file 1]. Patients with HIV-associated lipodystrophy were older and demonstrated dyslipidemia and a history of AIDS more frequently than patients without lipodystrophy. CD4 counts were higher (although not statistically significant) and the interquartile range of HIV viral loads was lower in subjects with lipodystrophy vs. subjects without lipodystrophy.

Among the 92 patients with clinical evidence of HIV-associated lipodystrophy, 15 (16%) had evidence of lipoatrophy only, 24 (26%) had evidence of lipohypertrophy only, and 53 (58%) demonstrated a mixed lipoatrophy/lipohypertrophy presentation. Patients with lipodystrophy reported physical changes for a duration of 28 (21, 48) [median(IQR)] months. Body image measures demonstrated significantly increased body image dysphoria and reduced body image-related quality of life among HIV-infected patients with lipodystrophy as compared to HIV-infected patients without lipodystrophy.

### Healthcare Utilization and Lipodystrophy Status

Clinical lipodystrophy status was associated with healthcare utilization outcomes [see Additional file 2]. In particular, the total number of healthcare encounters was significantly greater among patients with HIV-associated lipodystrophy as compared to those without. Clinic visits accounted for the majority of healthcare encounters, and patients with HIV-associated lipodystrophy attended more clinic visits than HIV-infected patients without lipodystrophy. In addition, admission to the hospital was more prevalent amongst patients with physician-defined HIV-associated lipodystrophy as compared to those without lipodystrophy, although associated length of stay and healthcare costs did not differ according to lipodystrophy status.

Healthcare expenditures paralleled healthcare use. Total healthcare costs were significantly greater among patients with HIV-associated lipodystrophy as compared to category counterparts; patients with HIV-associated lipodystrophy spent $1,718 more than HIV-infected patients without lipodystrophy during the year of observation. Similarly, costs associated with clinic visits were greater among patients with HIV-associated lipodystrophy than non-lipodystrophy patients, although this did not reach statistical significance.

Among patients with lipodystrophy, lipodystrophy subcategorizations (i.e. lipoatrophy only, lipohypertrophy only, or mixed presentation) were not significantly associated with healthcare utilization outcomes (p > 0.05). However, patients who reported a longer duration of lipodystrophy changes demonstrated a significantly greater number of healthcare encounters (23 (17, 33) vs. 13 (8, 25) visits, patients with lipodystrophy ≥ 28 months vs. patients with lipodystrophy <28 months, p = 0.01) and greater associated healthcare costs ($5,437 ($4,176, $9,716) vs. $3,034 ($1,918, $5,751), patients with lipodystrophy ≥ 28 months vs. patients with lipodystrophy <28 months, p = 0.01) compared to patients reporting lipodystrophy changes for a shorter period.

### Healthcare Utilization and AIDS and HCV Status

Healthcare utilization measures were also related to historical AIDS status in the study cohort. The total number of healthcare encounters was significantly greater among patients with a history of AIDS as compared to those without (17 (9, 26) vs. 12 (7, 20) visits, AIDS vs. non-AIDS, p = 0.01). The majority of these encounters were accounted for by scheduled clinic visits (17 (8, 25) vs. 11 (7, 20) visits, AIDS vs. non-AIDS, p = 0.01). Admission rates to the hospital were similar between patients with and without AIDS (p = 0.24).

Total healthcare expenditures were also greater among HIV-infected patients with historical AIDS as compared to patients without AIDS ($4,394 ($2,477, $8,138) vs. $2,670 ($1,365, $4,959), AIDS vs. non-AIDS, p = 0.0008). Clinic costs were also greater among patients with AIDS than non-AIDS patients ($3,014 ($1,424, $4,672) vs. $2,148 ($847, $3,408), AIDS vs. non-AIDS, p = 0.01). There were no differences in emergency room related costs between groups categorized by history of AIDS.

In contrast, presence of HCV did not affect healthcare utilization outcomes (p = 0.70 and p = 0.76, healthcare encounters and healthcare costs, respectively).

### Healthcare Utilization and Cardiovascular Risk

Healthcare utilization outcomes were associated with cardiovascular risk factors in the study cohort. Specifically, the total number of healthcare encounters was significantly greater among HIV-infected patients with hyperlipidemia and/or diabetes as compared to patient counterparts (18 (9, 25) vs. 11 (7, 20) visits, HIV-infected patients with hyperlipidemia and/or diabetes vs. HIV-infected controls, p = 0.003). Total healthcare expenditures were also greater among HIV-infected patients with hyperlipidemia and/or diabetes as compared to category comparisons ($4,373 ($2,266, $7,344) vs. $3,104 ($1,493, $5,405), HIV-infected patients with hyperlipidemia and/or diabetes vs. HIV-infected controls, p = 0.07), although this did not reach statistical significance. The total number of healthcare encounters (20 (11, 26) vs. 11 (7, 20) visits, HIV-infected patients with hypertension vs. HIV-infected normotensive patients, p = 0.001) and total healthcare expenditures ($4,764 ($2,719, $8,925) vs. $2,773 ($1,656, $5,102), hypertensive vs. normotensive HIV-infected patients, p = 0.002) were also significantly greater among HIV-infected patients with hypertension as compared to normotensive HIV-infected patients.

### Body Image measures and Healthcare Utilization outcomes in HIV-associated Lipodystrophy

Among patients affected by HIV-associated lipodystrophy, body image measures did not correlate with number of healthcare encounters (ρ = 0.09, p = 0.39 and ρ = -0.08, p = 0.46, BIQLI and SIBID-S, respectively). Similarly, in this subcohort, body image measures did not correlate with healthcare expenditures (ρ = 0.09, p = 0.40 and ρ = -0.05, p = 0.46, BIQLI and SIBID-S, respectively).

### Multivariate modeling

In multivariate regression analysis, the relationship between healthcare utilization (encounters) and lipodystrophy status (represented as lipodystrophy assessment score) remained significant controlling for age, sex, HIV viral load, CD4 count, and presence of cardiovascular risk or HCV disease [see Additional file 3]. The relationship between healthcare costs and lipodystrophy was also significant in multivariate modeling [see Additional file 3].

## Discussion

We performed an observational study among HIV-infected patients in care to determine whether lipodystrophy status affects healthcare services utilization. Our findings demonstrate that clinical somatic changes defined as lipodystrophy are associated with increased healthcare service utilization among HIV-infected patients despite improved HIV disease status measures.

Prior studies of hospitalization and outpatient services use among the HIV-infected community have demonstrated a significant relationship between worsening disease status (as represented by decreasing CD4 count and increasing HIV viral load) and increased health services utilization [11]. In addition, data from the National Ambulatory Medical Care Survey demonstrate that patients co-infected with HIV and HCV demonstrated increased hospitalization rates and hospital charges for HCV liver complications over the period of 1994 to 2001 [12]. However, we demonstrate that lipodystrophy is also a strong predictor of healthcare usage in analyses controlling for HIV disease status and HCV co-infection. Interestingly, in our study cohort, HIV viral load and HCV co-infection were not significantly related to healthcare utilization outcomes, and patients with lipodystrophy demonstrated higher CD4 counts and lower viral loads than comparison counterparts. Lipodystrophy has been shown to result from antiretroviral exposure and, in particular, is relatively common among persons taking highly active antiretroviral therapy (HAART) [2-4]. Although HAART has reduced morbidity and increased the life expectancy of persons infected with HIV [13,14], as reflected by improved disease measures (such as increased CD4 count and lower viral loads, as demonstrated by our cohort with lipodystrophy), the expected decrease in healthcare usage and healthcare expenditures has not been demonstrated [11]. One potential reason for this lack of improvement in healthcare utilization outcomes may be the notable prevalence of lipodystrophy in the HAART-exposed HIV-infected population (up to 80% in some studied populations [4]) and associated increases in healthcare usage by persons affected by HIV-associated lipodystrophy as demonstrated in our cohort.

The healthcare costs associated with lipodystrophy in our cohort was significant, even over the relatively short-time period of 1 year. The financial burden of HIV-associated lipodystrophy is substantial. The management of lipodystrophy remains a major challenge in HIV clinical care, given the lack of a currently available cure. Some therapies do hold promise; however, the cost of such therapies are often quite prohibitive for disadvantaged consumers [15]. Cosmetic options are available, but are not reimbursed by health insurers and thus must be paid out-of-pocket by often underprivileged consumers. In addition, clinical lipodystrophy is not only a cosmetic problem but also has been shown to co-exist with metabolic (glucose intolerance and hyperlipidemia [16]) and clinical (hypertension [17,18]) derangements associated with development of diabetes and cardiovascular disease, which incur significant healthcare costs [19-22]. In our cohort, patients with diabetes, dyslipidemia, and/or hypertension were found to have increased healthcare utilization and incur greater associated healthcare costs; however, the associations between diabetes and dyslipidemia (frequent cardiovascular risk factors associated with lipodystrophy) and healthcare outcomes did not remain significant after controlling for severity of lipodystrophy in multivariate analysis. Among non-HIV infected populations, visceral fat accumulation is associated with metabolic abnormalities and increased cardiovascular risk [23,24]. However, in our study, we did not demonstrate increased healthcare utilization among HIV-infected patients with lipohypertrophy-predominant lipodystrophy as compared to other lipodystrophy subtypes. Rather, we demonstrate increased healthcare use and cost among patients with any clinical HIV-associated lipodystrophy (inclusive of lipoatrophy and lipohypertrophy) as compared to HIV-infected patients without lipodystrophy. Healthcare utilization evaluation according to presence or absence of lipodystrophy is appropriate given that the data regarding visceral fat accumulation and metabolic abnormalities in the HIV-infected population is not as compelling as in non-HIV infected populations, and lipoatrophy also has been associated with metabolic and cardiovascular consequences [25].

Dramatic alterations in body appearances, such as is seen in HIV lipodystrophy, can distort the function and experience of the human body. Previously, we and others demonstrated that HIV lipodystrophy has significant negative effects on psychosocial well-being and quality of life [5,6]. In other populations with body cosmetic alterations, such as obese persons [26], psychological distress can lead to impairment of physical well-being and increased healthcare utilization. Although we demonstrate increased healthcare utilization and healthcare expenditures among patients affected by the HIV lipodystrophy syndrome as compared to HIV-infected patients without lipodystrophy, we did not demonstrate a direct association between healthcare utilization or costs and measures of body image dysphoria or body image-related quality of life.

Our findings are subject to a number of limitations. First, subjects recruited for the study were participants in a body image study and participants may have self-selected to participate in the study owing to their increased anxiety regarding body image. However, both patients with and without lipodystrophy were invited to participate in the study. Second, we retrieved healthcare utilization and cost data from one medical entity. However, this particular medical entity was the primary coordinating center for the HIV care for all study participants and therefore it is likely that participants did not seek care at other local medical offices. Nevertheless, we were not able to collect or account for any out-of-system charges. In this particular study, we chose to limit cost retrieval to one year only in order to reflect costs associated with the physical findings of lipodystrophy as determined by physician examination. Additional study will be needed to determine the longitudinal effects of lipodystrophy on healthcare utilization after initial diagnosis as compared to healthcare utilization previous to development of lipodystrophic body changes. Third, lipodystrophy was only assessed at a single time point; therefore, changes in body lipodystrophy on healthcare utilization over time could not be determined. Prior data has shown that lipodystrophy changes stabilize 18 to 24 months following initial development [27]. About half of subjects with lipodystrophy in this cohort had reported lipodystrophy of at least 28 months duration at the time of study, and subjects with a longer duration of lipodystrophy demonstrated greater healthcare utilization compared to those with a shorter duration of lipodystrophy. Thus, it would appear that our assessment may have actually underestimated the healthcare utilization of lipodystrophy subjects by including patients who had "early" lipodystrophy; the economic and healthcare service burdens associated with lipodystrophy may therefore be even greater than we have presented. Lastly, our findings of a significant relationship between HIV-associated lipodystrophy and healthcare utilization are correlative and not necessarily causal. We did attempt to control for potential confounders by including clinical and demographic variables in our analyses. Nevertheless, entered variables in our multivariate modeling of healthcare outcomes explained only a portion of the observed variability; therefore, lipodystrophy status accounts for but a portion of healthcare utilization and costs in our cohort. Alternatively, it is possible that unmeasured confounders explain the demonstrated relationship.

## Conclusion

In summary, we explored and provide evidence of the clinical and economic burden of HIV-associated lipodystrophy on healthcare utilization. Our study documents the association between healthcare use and severity of lipodystrophy using individual-level data, while taking age, sex, cardiovascular risk, HCV and AIDS status into consideration. Additional study is needed to further establish the clinical resource and financial burdens of lipodystrophy using data from a longer period.

## Methods

### Participants and Setting

181 HIV-infected subjects were recruited from an academic, multidisciplinary adult HIV clinic in San Diego for a study evaluating body image. Participants completed body image surveys and were assessed by a physician for the presence or absence of lipodystrophy. Details and main study outcomes of the body image study have been previously published [5,6] and are described below.

### Healthcare utilization outcomes

Healthcare utilization outcomes extracted from the medical record for each subject included number of ambulatory care (scheduled and urgent) visits, emergency room visits, hospitalizations and length of stay incurred over a 12-month period (10 months prior and 2 months after the study visit date) proximal to the assessment of lipodystrophy. In this study, our financial outcome for healthcare utilization was healthcare costs. Activity based costing was used to determine costs associated with patient care and included both direct (including laboratory testing, radiologic examinations, pharmacy, blood usage, respiratory care, nursing care, operating room and clinic room expenses, etc.) and indirect costs (overhead costs of plant maintenance, administration, medical records, human resources and information services). We chose to not evaluate healthcare charges, since charges often are subject to institutional marketing strategies and markup or markdown policy, and may vary by payor [28,29].

### Lipodystrophy assessment

A physical examination was performed by two study physicians to determine presence or absence of lipodystrophy. In their determination of the presence of lipodystrophy, study physicians were asked to assess 7 specific body areas for changes in fat distribution: face, neck and shoulder, arms, abdomen, buttocks and legs, and breasts using a 0-to-2 point bi-directional scale with 1/2-point increments to determine severity; the lipodystrophy assessment score was then determined by totaling the subscores of body changes from these 7 areas. While scale scores for each body area was noted in the positive (lipohypertrophy) or negative (lipoatrophy) direction, the lipodystrophy assessement score was calculated via addition of absolute value scores in each area. Thus, a higher lipodystrophy assessment score indicated a greater severity of lipodystrophy (inclusive of both lipohypertrophy and lipoatrophy) and ranged from 0 to 14. Between the two study physicians, agreement regarding absence or presence of lipodystrophy was 91% (both assessed 11 randomly selected subjects)[5].

### Questionnaires

The Body Image Quality of Life Inventory (BIQLI) is a clinical assessment of how an individual's body image impacts his or her life. The BIQLI uses a 7-point response format ranging from very negative (-3) to very positive (+3) effects of body image on 19 life domains [30]. The nineteen-item BIQLI is internally consistent and has been demonstrated to converge significantly with multiple measures of body-image evaluation as well as with body mass. The BIQLI is valuable for quantifying how persons' body image experiences affect a broad range of life domains, including sense of self, social functioning, sexuality, emotional well-being, eating, exercise, grooming, etc. The BIQLI is scored as an average numeric score of the 19 items where a more negative score reflects a more negative body image.

The Situational Inventory of Body-Image Dysphoria (SIBID) is an assessment of the frequency of negative body-image emotions across specific situational contexts. This inventory asks respondents how often they experience body-image dysphoria or distress (according to a numeric range of 0 (never) to 4 (always)) in each of 48 identified situations-including both social and nonsocial contexts and activities related to exercising, grooming, eating, intimacy, physical self-focus, and appearance alterations. Research has confirmed that this is an internally consistent, stable, and convergently valid measure of body-image affect that is responsive to body-image therapy. Recently, a 20-item short form of the SIBID has been validated and found to correlate highly (r > .95) with the original longer version [31]. The short form of the SIBID (SIBID-S) was used in this study. The SIBID-S is scored as the numeric mean score of its 20 items where a higher score is associated with increased body image dysphoria.

### Other data

Demographic data were also collected. CD4 count, HIV viral load, and duration of lipodystrophy changes at the time of the lipodystrophy evaluation were extracted from the medical record. Diagnoses of hypertension, dyslipidemia, diabetes, and HCV were extracted from the medical record via associated ICD-9 codes and/or laboratory results.

### Response Coding

Healthcare utilization outcomes, body image questionnaire scores, age, CD4 count and HIV viral load were not modified. Racial response categories included: white, black, Hispanic, Asian, or other; for the purposes of analysis, these groups were collapsed according to Caucasian or non-Caucasian origin. Sex was coded as male or female. Lipodystrophy status was coded as present or absent. Among patients with lipodystrophy, further categorization of lipodystrophy according to lipodystrophy assessment scores was performed into lipoatrophy only, lipohypertrophy only, and mixed lipoatrophy/lipohypertrophy groups. Patients with lipodystrophy were also categorized according to duration of lipodystrophy changes (<28 months vs. ≥ 28 months). HIV disease status was coded as having ever met AIDS diagnostic criteria or not (i.e. history of AIDS or not). Diagnoses of hypertension, dyslipidemia, diabetes, and HCV were coded as present or not.

### Statistical Analysis

Healthcare utilization outcomes and other selected measures were compared according to presence or absence of lipodystrophy, lipodystrophy subcategories (among patients with HIV-associated lipodystrophy only), history of AIDS, and history of cardiovascular risk or HCV using chi-square statistics for categorical variables and the Wilcoxon rank sum test for continuous variables. Spearman's correlation analysis was used to determine potential associations between body image measures and healthcare utilization outcomes among the subpopulation affected by HIV-associated lipodystrophy. Multivariate linear regression analyses were then applied to identify predictors of healthcare utilization outcomes after adjusting for clinical variables (age, sex, HIV viral load, CD4 count, and presence of cardiovascular risk or HCV). Clinical variables entered into the regression models were selected owing to their known associations with lipodystrophy, HIV disease, and/or healthcare outcomes. For multivariate regression methods and for correlation analyses, we transformed some variables to improve the symmetry of their distributions. For example, log_10 _transformation improved the symmetry of CD4 count and HIV viral load distributions. Statistical analyses were performed using JMP 5.0 (SAS Institute, Inc., Cary, NC).

## Competing interests

The authors declare that they have no competing interests.

## Authors' contributions

JSH conceived of the study, participated in its design and performance, and drafted the initial manuscript. KB and SF participated in study performance and data collection. WCM and DL helped to draft and revise the manuscript. All authors read and approved the final manuscript.

## Supplementary Material

Additional File 1 **Table 1**. Population Demographics. Click here for file

Additional File 2 **Table 2**. Healthcare utilization outcomes according to lipodystrophy status. Click here for file

Additional File 3 **Table 3**. Multiple Linear Regression Analyses on Healthcare Utilization Outcomes and Selected Patient Characteristics. Click here for file
