# Supplementary material for: The impact of HIV-associated lipodystrophy on healthcare utilization and costs
Source: AIDS Res Ther. 2008 Jul 1;5:14. doi: 10.1186/1742-6405-5-14 (PMC2478721; doi:10.1186/1742-6405-5-14)
Supplement: Additional File 1 — Table 1. Population Demographics. [file 1742-6405-5-14-S1.pdf]

TABLE 1. Population Demographics

	Entire Cohort (n=181)	HIV + with Lipodystrophy (n=92)	HIV+ without Lipodystrophy (n=89)	p-value between Lipodystrophy groups
Age (years)*	43 (38, 50)	44 (38, 50)	41 (36, 46)	0.03[¥]
Race	32% Hispanic,<br>9% black,<br>52% white,<br>2% Asian,<br>5% other	34%<br>9%<br>52%<br>2%<br>3%	29%<br>10%<br>52%<br>2%<br>7%	0.83[Φ]
Sex (Male: Female)	128: 53	60: 32	68: 21	0.10[Φ]
CD4 count (cells/μL)*	463 (262, 650)	496 (317, 678)	421 (242, 641)	0.06[¥]
Log_{10} HIV viral load (copies/mL)*	2.6 (2.6, 3.4)	2.6 (2.6, 2.6)	2.6 (2.6, 3.8)	0.04[¥]
Lipodystrophy Assessment Score*	1.5 (0, 3.0)	3.0 (2.0, 4.5)	0.5 (0, 1.0)	<0.0001[¥]
SIBID-S score*		2.1 (1.4, 2.9)	1.3 (0.6, 2.0)	<0.0001[¥]
BIQLI score*		-0.5 (-1.2, 0.4)	0.4 (-0.4, 1.5)	<0.0001[¥]
Hypertension (N (%))	77 (43%)	43 (47%)	34 (38%)	0.25 [Φ]
Diabetes (N (%))	36 (20%)	20 (22%)	16 (18%)	0.53 [Φ]
Dyslipidemia (N (%))	102 (56%)	59 (64%)	43 (48%)	0.03 [Φ]
HCV (N (%))	22 (12%)	13 (14%)	9 (10%)	0.41 [Φ]
AIDS (N (%))	102 (56%)	59 (64%)	43 (48%)	0.03[Φ]

*Expressed as median (interquartile range).

[¥]p-value by Wilcoxon rank sum test. [Φ]p-value by chi-square statistics.
