# Supplementary material for: The impact of HIV-associated lipodystrophy on healthcare utilization and costs
Source: AIDS Res Ther. 2008 Jul 1;5:14. doi: 10.1186/1742-6405-5-14 (PMC2478721; doi:10.1186/1742-6405-5-14)
Supplement: Additional File 2 — Table 2. Healthcare utilization outcomes according to lipodystrophy status. [file 1742-6405-5-14-S2.pdf]

Table 2. Healthcare utilization outcomes according to lipodystrophy status.

**Healthcare utilization variable**	**HIV + with Lipodystrophy (N=92)**	**HIV+ without Lipodystrophy (N=89)**	**p-value**
**Total Healthcare Encounters**	**19 (10, 26)**	**11 (7, 20)**	**0.0002**
**Total Healthcare Costs**	**$4,731 ($2,427, $8,242)**	**$3,013 ($1,493, $5,047)**	**0.007**
**Clinic Visits**	**18 (9, 26)**	**10 (6, 19)**	**0.0003**
**Clinic Costs**	$2,841 ($1,274, $4,478)	$2,193 ($939, $3,653)	0.06
**Emergency Room Visits**	0 (0, 1)	0 (0, 0)	0.20
**Emergency Room Costs**	$0 ($0, $469)	$0 ($0, $0)	0.20
**Hospital Admission during 12 month period (%)**	**21%**	**9%**	**0.03¥**
**Length of Stay (days)***	3 (2, 8)	3 (1, 5)	0.21
**Hospital Admission Costs***	$4,668 ($0, $8,163)	$4,775 ($1,608, $9,508)	0.67

Data reported as median (IQR) unless otherwise specified. p-value by Wilcoxon rank sum test unless otherwise specified.

¥p-value by chi-square analysis. *Data from hospitalized patients only.
