# Supplementary material for: The impact of HIV-associated lipodystrophy on healthcare utilization and costs
Source: AIDS Res Ther. 2008 Jul 1;5:14. doi: 10.1186/1742-6405-5-14 (PMC2478721; doi:10.1186/1742-6405-5-14)
Supplement: Additional File 3 — Table 3. Multiple Linear Regression Analyses on Healthcare Utilization Outcomes and Selected Patient Characteristics. [file 1742-6405-5-14-S3.pdf]

TABLE 3. Multiple Linear Regression Analyses on Healthcare Utilization Outcomes and Selected Patient Characteristics.

**Dependent Variable: Healthcare Utilization (Healthcare Visits over study period), r^2=0.13, whole model p=0.003**			
Variable	*Estimate*	*Standard Error*	*p-value*
**Lipodystrophy Assessment Score**	**1.29**	**0.46**	**0.006**
**CD4 count**	**-5.29**	**2.58**	**0.04**
HIV viral load	-1.16	1.09	0.29
Sex (female)	1.36	1.03	0.19
Age	0.02	0.10	0.85
Presence of diabetes or dyslipidemia (no)	1.05	0.93	0.26
Presence of hypertension (no)	1.06	0.96	0.27
HCV (not infected)	0.73	1.27	0.57
**Dependent Variable: Healthcare Costs (Healthcare Costs incurred over study period), r^2=0.09, whole model p=0.03**			
Variable	*Estimate*	*Standard Error*	*p-value*
**Lipodystrophy Assessment Score**	**617**	**290**	**0.03**
**CD4 count**	**-3753**	**1618**	**0.02**
HIV viral load	-858	681	0.21
Sex (female)	-382	648	0.56
Age	-21	62	0.74
Presence of diabetes or dyslipidemia (no)	17	584	0.98
**Presence of hypertension (no)**	**1206**	**600**	**0.046**
HCV (not infected)	-242	798	0.76

Note: The reference group is presented after each variable in parentheses.
